# MESIA: multi-epigenome sample integration approach for precise peak calling

**DOI:** 10.1038/s41598-023-47948-2

**Published:** 2023-11-27

**Authors:** Seung Gwa Park, Woo-Jin Kim, Jae-I Moon, Ki-Tae Kim, Hyun-Mo Ryoo

**Affiliations:** 1 Department of Molecular Genetics & Dental Pharmacology, School of Dentistry and Dental Multiomics Center, Dental Research Institute, Seoul National University, Seoul, South Korea; 2 Epigenetic Regulation of Aged Skeleto-Muscular System Laboratory, School of Dentistry and Dental Research Institute, Seoul National University, Seoul, South Korea

**Keywords:** Bioinformatics, Software

## Abstract

The assay for transposase-accessible chromatin with sequencing (ATAC-seq) is the most widely used method for measuring chromatin accessibility. Researchers have included multi-sample replication in ATAC-seq experimental designs. In epigenomic analysis, researchers should measure subtle changes in the peak by considering the read depth of individual samples. It is important to determine whether the peaks of each replication have an integrative meaning for the region of interest observed during multi-sample integration. We developed multi-epigenome sample integration approach for precise peak calling (MESIA), which integrates replication with high representativeness and reproducibility in multi-sample replication and determines the optimal peak. After identifying the reproducibility between all replications, our method integrated multiple samples determined as representative replicates. MESIA detected 6.06 times more peaks, and the value of the peaks was 1.32 times higher than the previously used method. MESIA is a shell-script-based open-source code that provides researchers involved in the epigenome with comprehensive insights.

## Introduction

Next-generation sequencing (NGS) growth has paved the way for epigenetics analysis of post-transcriptional changes and various related analysis methods have been developed. Recently, many studies have shown higher quality results by combining^1^ transcriptomic analysis through RNA-seq^2^ and chromatin accessibility analysis^3–5^.

Among the many methods for measuring chromatin accessibility, the assay for transposase-accessible chromatin with sequencing (ATAC-seq), which requires relatively little time and a smaller amount of samples for analysis, is widely used^4^. Researchers supplement the lack of consistency by including multi-sample replication in experimental designs. In this process, it is important to integrate the information from biological replicates. In epigenome analyses, including ATAC-seq, which require the extraction of peaks from individual samples to measure subtle changes in specific regions, require integrated analysis to determine whether the results of the measured replication are consistent. Furthermore, the measurement of peak epigenome levels via NGS is accompanied by the process of extracting significant signals compared to background signals using complex algorithms, such as hidden Markov models^6^. Therefore, it is important to determine an optimal peak within a region of interest to ensure the reliability of the research results by measuring the similarity of individual samples rather than by simple merging.

Currently, methods used for ATAC-seq replication integration can be divided into two categories. One is the length overlap-based approach and the other is the statistical-based approach^7^. The length overlap-based approach is a method of determining the same peak based on the degree of overlap between the peaks of each replication, On the other hand, a statistical-based approach uses the reliable boundaries of reproducibility through statistical parameters to determine the reproducibility of peaks. In the case of the length-overlap-based approach, integration is possible regardless of the number of replications by focusing on speed and convenience; However, because reproducibility is derived based on the degree of overlap in length, the process of confirming statistical significance is not carried out. In the case of a statistical-based approach, replication integration is performed after determining the presentation and reproducibility based on the statistical background; however, only two replications are possible^7^. That means, no statistical background-based methods that are not significantly constrained by the number of replicates exist.

Here, we developed a novel replication integration method called Multi-Epigenome Sample Integration Approach for Precise Peak Calling (MESIA). MESIA determines replication with high representativeness and reproducibility in multi-sample replication and then integrates them to extract more robust and less likely false positive peaks. For researchers whose work may have been difficult to analyze due to a lack of computational background, a shell-script-based open-source code was disclosed to GitHub (https://github.com/ERASMUSlab/MESIA).

## Materials and methods

### Assay for transposase-accessible chromatin using ATAC-seq workflow

**Figure 1:**
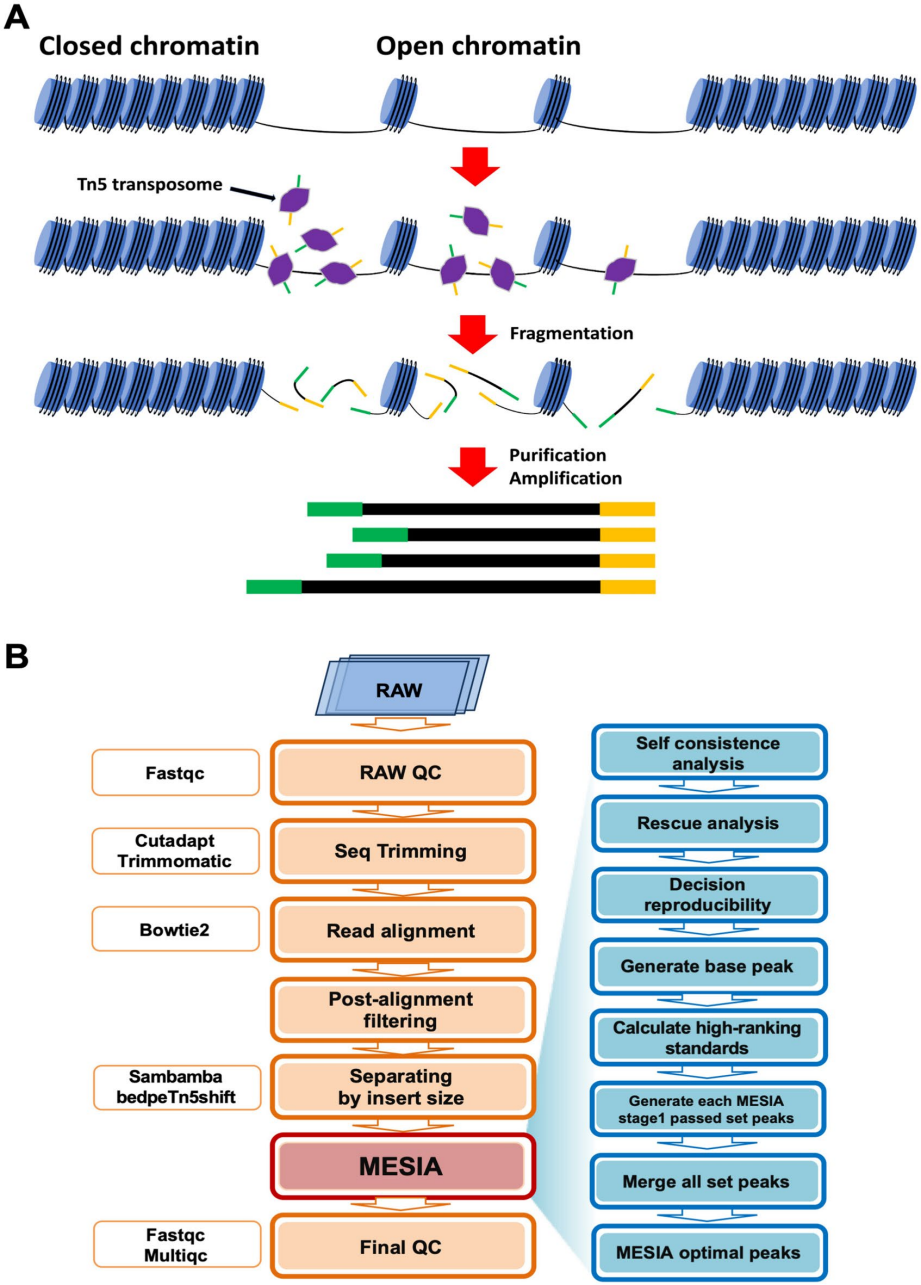
Assay for transposase-accessible chromatin using ATAC-seq workflow. (**A**) Basic concept and principle of ATAC-seq. (**B**) Generalized ATAC-seq preprocessing pipeline. MESIA is a tool that takes a BAM file as input and determines and integrates the reproducibility of replication's peak. It first checks the bias within the replication through self-consistency analysis. It then checks the reproducibility between replications through rescue analysis. Finally, it generates optimal peaks based on the Q value and the peak point of the base peak. It repeats this process for each replication and merges the results to produce MESIA optimal peaks.

The GM12878 human lymphoblastoid cell line^4^ was mainly used to verify the pipeline for ATAC-seq (Fig. 1A), and mouse embryonic fibroblasts^8^ (MEFs) were also used to verify the pipeline on other datasets. Sequence trimming was performed using Cutadapt^9^ and Trimmomatic^10^. Subsequently, the filtered fastq was aligned to the hg19 genome assembly for GM12878 and mm10 for MEFs using Bowtie2^11^. The generated BAM file was sorted, filtered by MAPQ quality, and PCR duplicates and mitochondrial reads were removed using Samtools^12^, and multi-mapping control was performed using assign_multimappers.py^13^, provided by Encode. Finally, the filtered BAM file was divided by histone status using Sambamba and bedpeTn5shift^14^. MESIA takes BAM files as input and uses MACS2^15^(v2.2.7.1) for peak calling. Through a series of processes, MESIA determines and integrates the reproducibility of multiepigenomic samples. (Fig. 1B).

### RNA-sequencing data processing

RNA-seq data obtained from two biological replicates were analyzed on the GM12878 human lymphoblastoid cell line^4^ and three biological replicates were analyzed on the MEFs^8^. The median read length was 101 bp. The reads were aligned hg19 and mm10 using Bowtie2^11^(v2.2.5), Samtools^12^(v1.13), Bamtools^16^(v2.5.1), Bedtools^17^(v2.30.0), Biobambam^18^(v2.0.183), Cutadapt^9^(v4.0), and Sambamba^19^(v0.8.2). Expression calling was performed using Salmon^20^(v1.7.0) and Kallisto^21^(v0.46.2).

### Multi-epigenome sample integration approach for precise peak calling (MESIA) workflow and pseudocode

**Figure 2:**
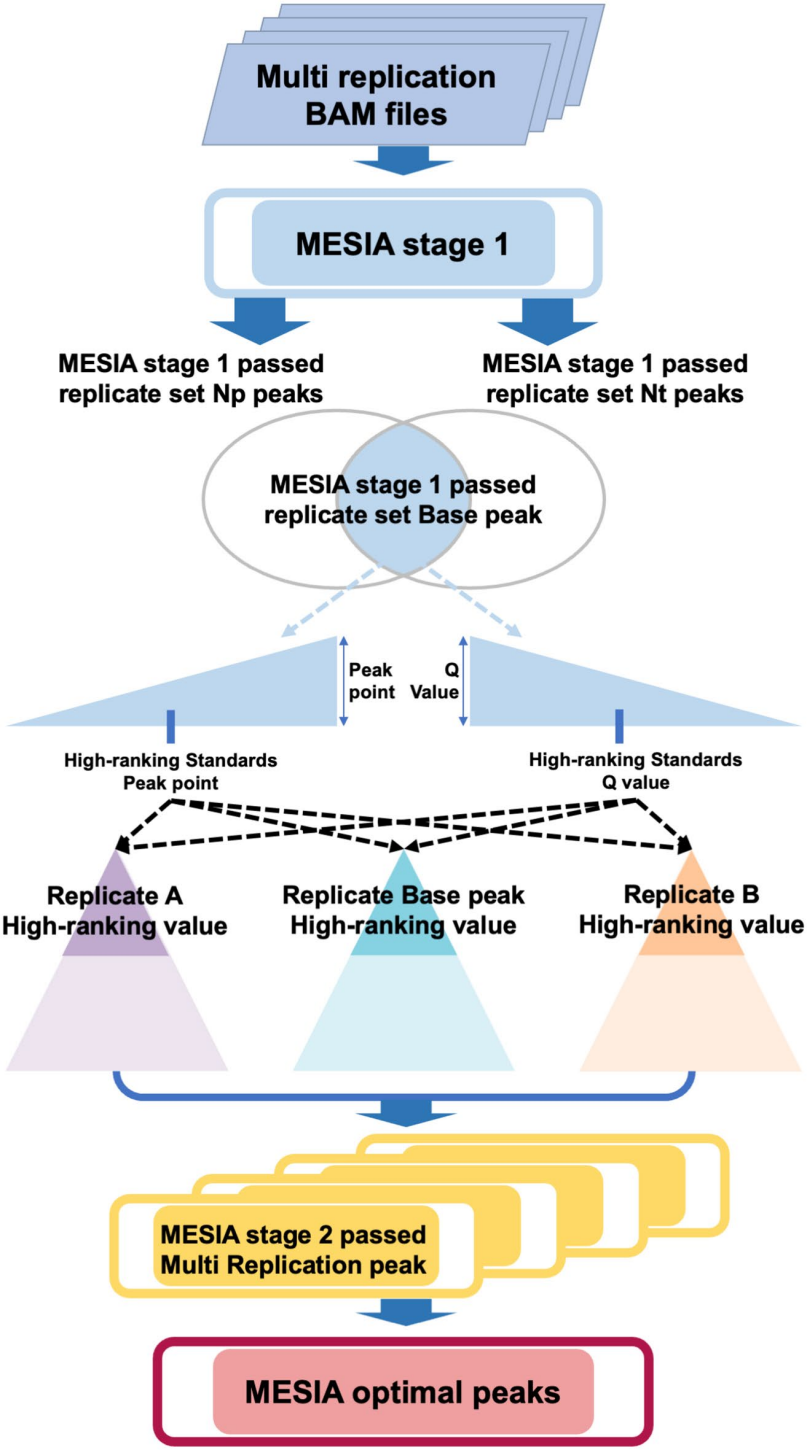
Multi-Epigenome Sample Integration Approach for precise peak calling (MESIA) workflow. MESIA is executed using a two-stage strategy, where stage1 determines the reproducibility of multi-epigenome samples, and stage2 merges reproducible replications. A hypothetical replication A and replication B were grouped together to represent a replication set. If the replication set passes MESIA stage 1, the base peak of the replication set is derived and the replication set base peak, replication A, and replication B are filtered using the median of the peak point and Q value of the base peak. The high-ranking peak sets obtained in this way are merged to produce MESIA stage 2 passed multi replication peak. After repeating this process with several other replication sets and merging them, MESIA optimal peak is produced.

MESIA is a method of selecting only reproducible replications in consideration of representativeness in multi-replication ATAC-seq and then deriving an optimal peak within replications (Fig. 2).

1 $${R}_{ij}=\left\{\begin{array}{c}1 \left({Nt}_{ij}/{Np}_{ij}\le 2\right)\\ 0 \left({Nt}_{ij}/{Np}_{ij}>2\right)\end{array}\right.$$

2 $$SubS=\left\{\begin{array}{c}1 \left(Rep-pseudo \;rep1/rep2\le 2\right)\\ 0 \left(Rep-pseudo \;rep1/rep2>2\right)\end{array}\right.$$

3 $${MS1pass}_{ij}=P\left({R}_{ij}=1|\left( {SubS}_{i} + {SubS}_{j}=2\right)\right)$$

Subsequently, it goes through MESIA stage 1 based on the IDR^22^. MESIA stage 1 is process of determining whether two replications are reproducible to each other, indicating that Nt called “peaks consistent between true replicates” and Np called “peaks consistent between poured pseudoreps” are less than twice that of each other. Furthermore, an analysis is conducted to examine potential biases within the sample by generating pseudo-replicates for each replicate through random read splitting. These conditions are reflected in constraints (1) and (2), where the variables i and j can range from 1 to the number of replications being considered as inputs. Constraint (3), MS1pass, refers to the replication set that passed MESIA Stage 1, which is the process of deriving a replication set with reproducibility based on the previous analysis.

4 $${SigBP}_{ij} = {\int }_{BPpp[(n+1)/2]}^{BPpp[n]}BP d(BPpp) \cap {\int }_{BPqv[m]}^{BPqv[(m+1)/2]}BP d(BPqv)$$

5 $$\mathrm{SigSP} = {\int }_{SPpp\left[\left(n+1\right)/2\right]}^{SPpp\left[n\right]}SP d\left(SPpp\right) \cap {\int }_{SPqv\left[m\right]}^{SPqv[(m+1)/2]}SP d\left(SPqv\right)$$

6 $${MS2pass}_{ij} = {SigBP}_{ij} \cup {SigSP}_{i} \cup {SigSP}_{j}$$

MESIA stage 2 uses Nt and Np of the replication set with the reproducibility selected in MESIA stage 1. The intersection of Nt and Np is called the base peak of the replication set, and MESIA stage 2, which passes the multi-replication peak, is obtained using the base peak. The base peak is labeled as BP in constraint (4). Similarly, within the MESIA Stage 1 passed replication set, the peak of a single replication is denoted as SP in constraint (5). High-ranking standards for calculating constraint (4, 5, 6) include the peak point, the peak point, distance from the start site to the highest signal site of the peak, and the Q value, which indicates statistical significance. Since base peak have already passed MESIA stage 1, the significant criteria for peak point and Q values were set to the medians of the base peak. This process is described in constraints (4) and (5), where the variable n represents the position of the largest value when sorting the peak points in descending order, and similarly, the variable m represents the position of the smallest value when sorting the q values in ascending order. Using these high-ranking standards, we derived a robust peak from the two replicates in the replication set. Constraint (6), MS2pass, refers to the MESIA Stage 2 passed peak.

7 $$MESIA \;optimal = \sum_{i,j = 1}^{i, j = max}{MS2pass}_{ij}$$

**Figure 3:**
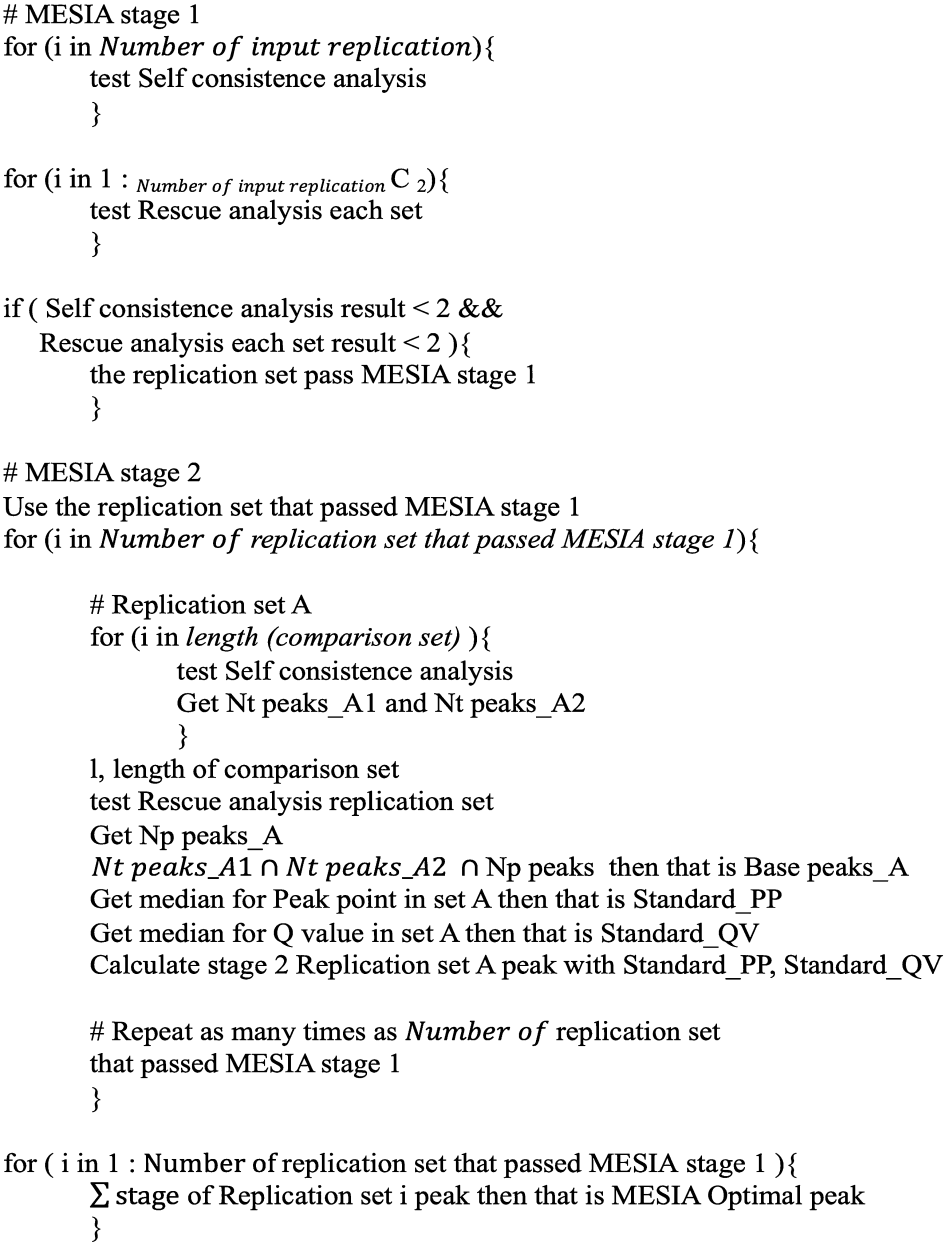
MESIA Pseudocode. Pseudo-code of the MESIA algorithm.

Finally, the MESIA optimal peaks are derived by merging the robust peaks of all replications sets that have passed through MESIA Stage 2. This process is described in constraint (7). Each step mentioned above are summarized into pseudocode (Fig. 3). MESIA stage 1 consists of self-consistency and rescue analysis, which are represented by constraints (2) and (1), respectively. These constraints detect bias within each replication and assess reproducibility between different replications. The base peaks of the replications that pass MESIA stage 1 are found using constraint (3). MESIA stage 2 is the stage that creates optimal peaks using the base peaks from MESIA stage 1. The process of filtering peaks using the peak point and Q value of the base peaks is represented by constraints (4–5). The process of combining these peaks to create optimal peaks is represented by constraints (6–7).

### Comparative algorithms related to length-based approaches and statistical-based approaches

**Figure 4:**
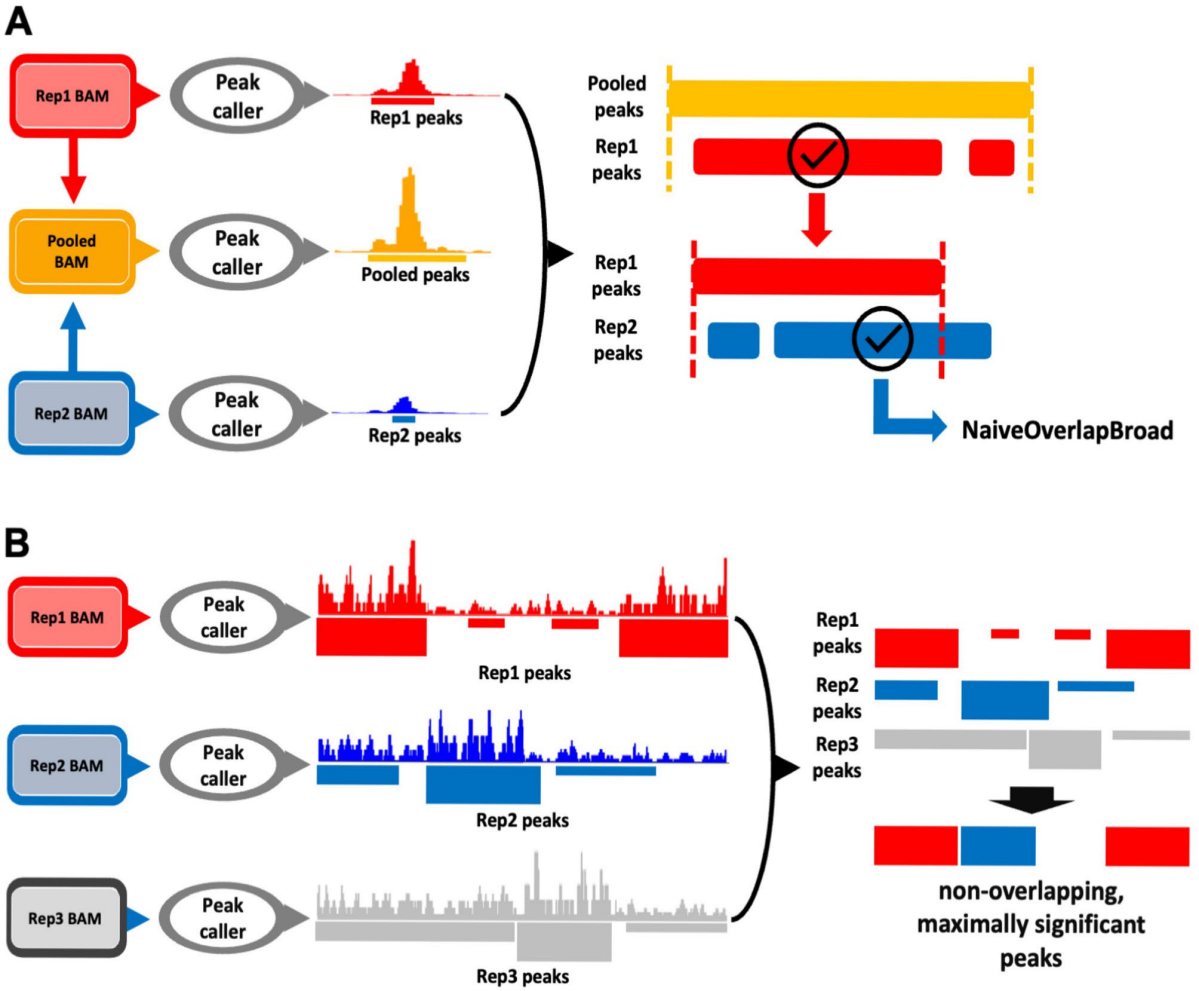
Comparative algorithms related to length-based approaches and statistical based approaches. (**A**) a schematic diagram of NAIVE, the length-based approaches algorithm. (**B**) a schematic diagram of NOMS, union approaches algorithm.

Other methods were used for comparison to measure the performance of MESIA. First, the method representing the length overlap-based approach uses NaiveOverlapBroad^23^ (NAIVE) (Fig. 4A). NAIVE is a method of pooling all replications to be integrated and then selecting only peaks that overlap by more than half in each replication. This method is not limited to the number of replicates; however, there is a risk of integration between replicates that lack statistical evidence and reproducibility. Statistical-based approaches include IDR^24^ and ChIP-R^25^. IDR is a statistical method that is widely used in two-replication integrations and considers the reproducibility between replications; however, it has the disadvantage of not being able to perform multi-sample integration. ChIP-R can perform multi-sample integration, but it does not consider the reproducibility between replications and has the disadvantage of not being robust due to overlapping replication parameters selected by the user. Finally, the non-overlapping maximum signal peak method^26^ (NOMS) (Fig. 4B) is an integration of the concept of a combination that could maximize the scope the scope of the optimal peak. In terms of stringency, ChIP-R, a statistical-based approach, used several default statistical values as thresholds. The ranking method was *p*-value, and the cut-off value for the set of reproducible peaks was 0.05. Length overlap-based approaches (NAÏVE and NOMS, Fig. 4) did not use statistical values. NAÏVE selected peaks that overlapped by more than half in each replication, while NOMS integrated the concept of a combination to improve accuracy.

### Compiling a basic simulation sample set

**Figure 5:**
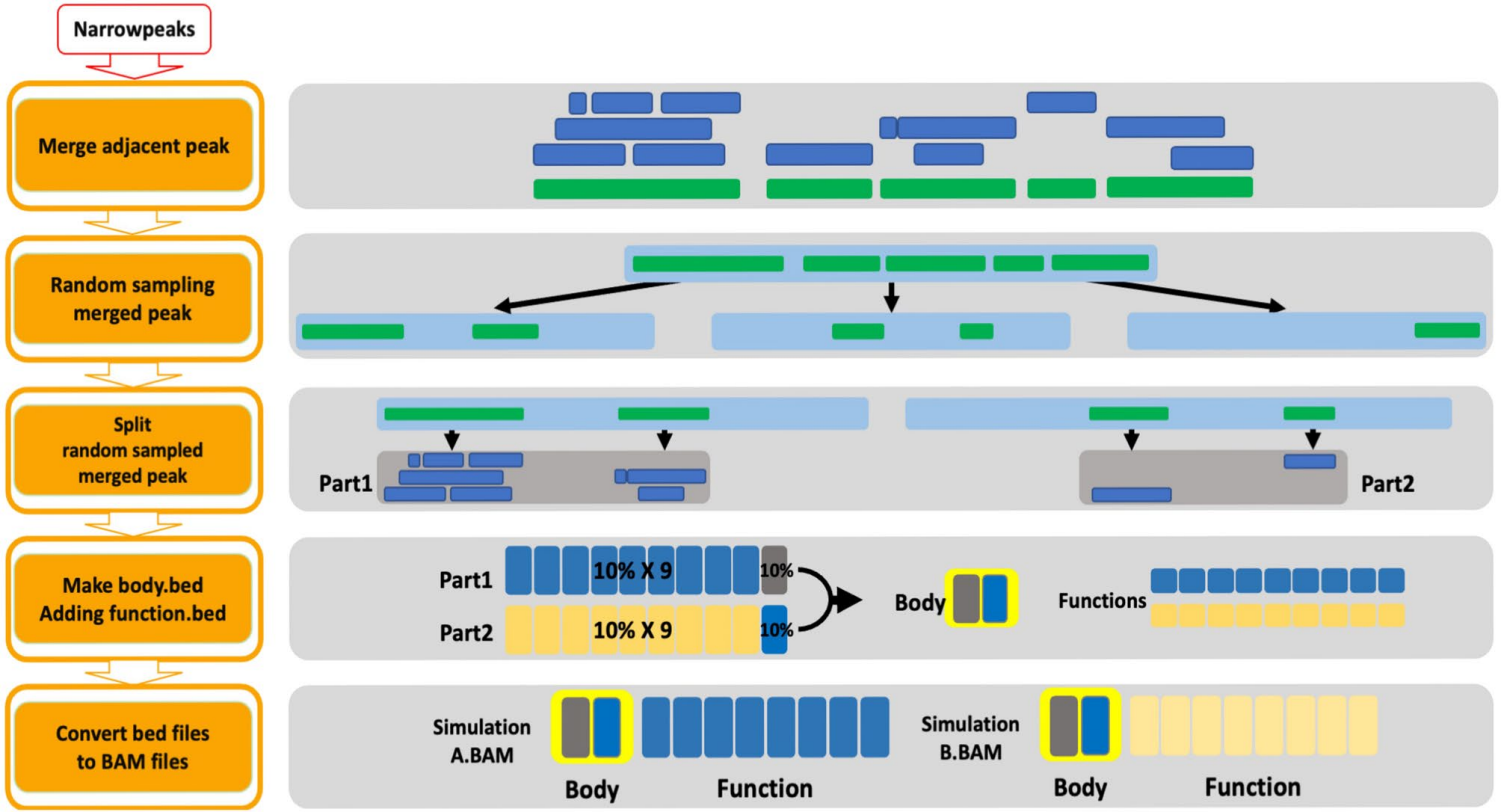
Compiling simulation sample set. Process of compiling a simulation sample set to determine the performance difference between MESIA and the compared algorithms. To ensure nonreproducibility between the simulation sets, samples A and B were created by dividing the peaks obtained from a single ATAC sample.

We needed a simulation sample with 7 characteristics to perform this simulation. Simulation samples A and B are simulation samples that are not reproducible with each other. Using the GM12878 replication1 ATAC narrowPeaks file, samples A and B with different trends were created. First, adjacent peaks were combined to form small set. Subsequently, the adjacent peaks were randomly sampled and divided into three large set. Select two large set out of three, and then divide the adjacent peak back into a narrowPeaks form. No matter how different the samples tend to be, there are basically shared peaks which were called body peaks. On the other hand, there must also be peaks that distinguish A and B, which we call function peaks. The body peaks and function peaks are combined to form A and B (Fig. 5). The reproducibility of samples A and B produced in this way is shown according to the reading of the body and function peaks. There was a phenomenon in which the number of Nt stabilized when the read of the function peak increased by more than a certain ratio. In consideration of this, sample A and sample B were made based on body peak reads 0.8 M and function peak reads 3.2 M by choosing a case where the result of rescue analysis was greater than 2 (Table 1). After that, the remaining 5 simulation samples were created using simulation sample A, B and the remaining one peak set that was not used to create simulation samples A and B. Simulation samples C and D are samples that do not have reproducibility for A and B. Among them, the simulation sample that is less related to A and B was made as C using the remaining one large set that was not used to create simulation samples A and B. And simulation sample D was randomly sampled at 10% each of sample A and B. Finally, simulation samples E, F, and G are samples that have reproducibility for A and B. The degree of reproducibility for A and B increases in the order of E, F, and G. Simulation samples E, F, and G were randomly selected at 30%, 50%, and 60% of samples A and B, respectively (Supplementary Fig. 9).

**Table 1:** Summary of intermediate results during compilation of simulation sample set.

Body reads (M)	Function reads (M)	Np peaks	Nt peaks	Rescue analysis result	Decision
0.8	0.4	9445	47,313	5.009	Unstable
	0.8	12,238	47,313	3.866	Unstable
	1.2	14,904	47,314	3.174	Unstable
	1.6	17,751	47,313	2.665	Unstable
	2	20,247	47,313	2.336	Unstable
	2.4	23,036	47,314	2.053	Unstable
	2.8	25,882	47,314	1.828	Similar replication set
	3.2	28,841	10,953	2.633	Different replication set
1.6	0.4	16,175	94,552	5.845	Unstable
	0.8	18,517	94,552	5.106	Unstable
	1.2	21,318	94,554	4.435	Unstable
	1.6	23,956	94,554	3.946	Unstable
	2	26,794	94,554	3.5289	Unstable
	2.4	29,734	21,901	1.357	Similar replication set
	2.8	32,495	21,901	1.487	Similar replication set
2.4	0.4	22,105	141,862	6.417	Unstable
	0.8	25,049	141,864	5.663	Unstable
	1.2	24,856	141,862	5.092	Unstable
	1.6	30,551	32,672	1.069	Similar replication set
	2	33,616	32,672	1.028	Similar replication set
	2.4	36,190	32,672	1.107	Similar replication set
3.2	0.4	29,193	188,891	6.470	Unstable
	0.8	31,690	43,977	1.387	Similar replication set
	1.2	34,510	43,977	1.274	Similar replication set
	1.6	37,067	43,977	1.186	Similar replication set
	2	39,859	43,977	1.103	Similar replication set

M, million units; Np, peaks consistent between poured pseudoreps: Nt, peaks consistent between true replicates.

### Compiling a different state's simulation sample set that highlights the difference in reproducibility

To evaluate the performance differences between the compared algorithms based on the reproducibility of the simulation sets, we defined three different states: standard, hard, and soft. Specifically, in the standard state, we used simulation set F, which has a 50% overlap with either simulation set A or B. However, in the hard state, we used simulation set E, which has only a 30% overlap. Conversely, in the soft state, we used simulation set G, which has a 60% overlap.

### Compiling a different state's simulation sample set that highlights the number of similar replications

To evaluate the performance differences of the compared algorithms based on the number of similar replications of the simulation sets, we defined three different states: strong, normal, and weak. In the normal state, we used cases of three and four replication sets that passed through MESIA stage1. In the strong state, we used cases of five and six replication sets that passed through MESIA stage1. Finally, in the weak state, we used the case of one and two replication sets that passed through MESIA stage1.

### Calculating peak distribution and gene expression

Peaks formed in the promoter region derived from each method were used. Performance was compared based on the difference in the degree of gene expression related to the peak formed in the promoter. Gene expression information was derived from GM12878 polyA- and RNA-seq. it was aligned to the hg19 genome assembly.

## Results

### Differences in peak contents between comparative algorithms

**Figure 6:**
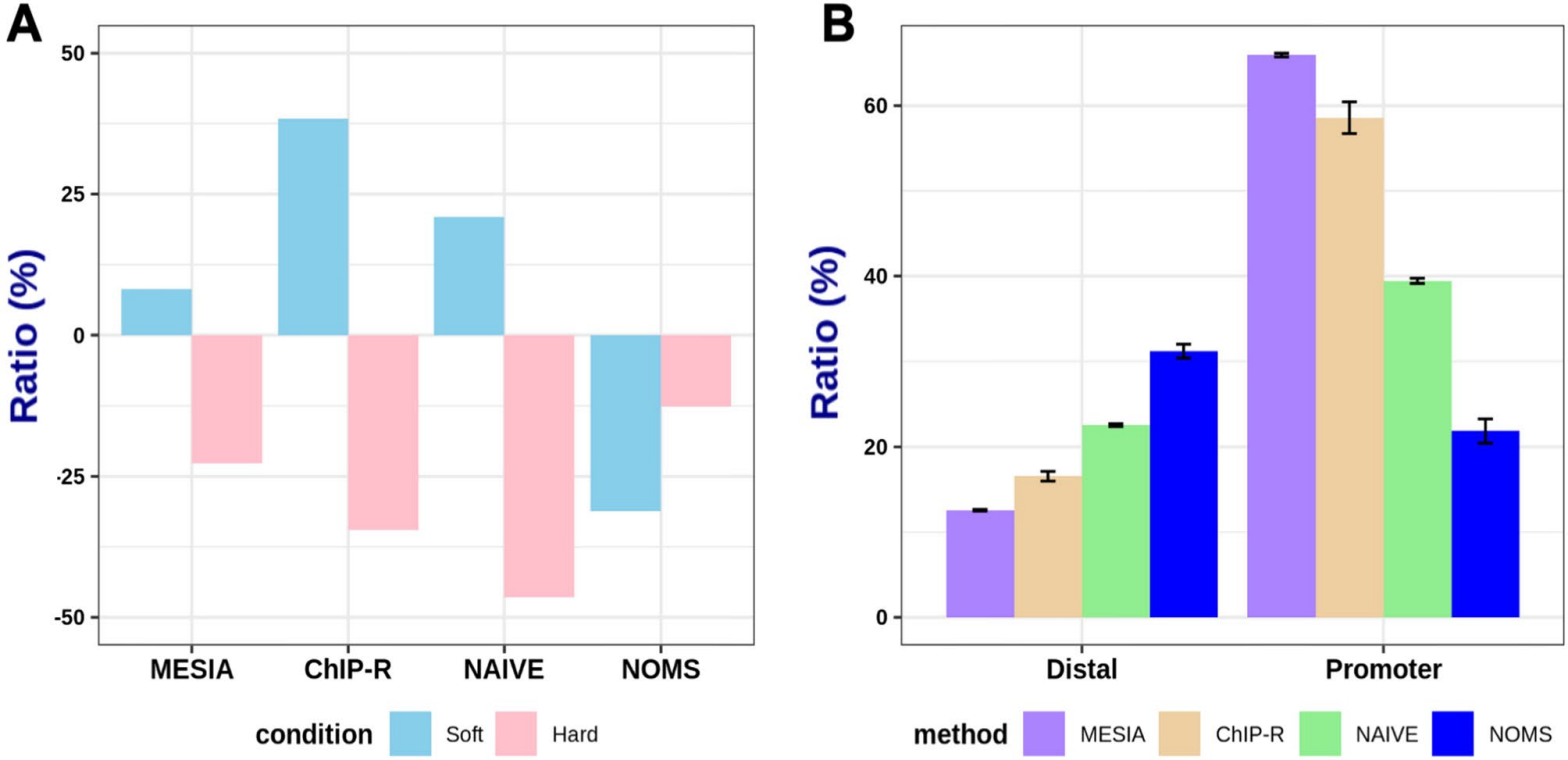
Differences in peak contents between comparative algorithms, depending on a degree of reproducibility. (**A**) Bar plot of difference according to condition across methodologies. In a standard state, we use simulation set 50% overlap with either simulation set A or B. In a hard state, use simulation set 30% overlap. Last, in a soft state, we use simulation set 60% overlap. Compared to the number of peaks in the standard state, an increase and decrease of peak counts in the soft state are represented by pink bars, while the hard state is represented by sky blue bars in the same way. (**B**) Comparative bar plot between distal and promoter regions. Under the hard state conditions, the ratio of genes that form peaks in both the distal intergenic and promoter regions were determined using the optimal peaks of each comparative algorithm. region was set to be 3 KB away from the transcription start site (TSS).

When comparing the changes in the number of peaks between the soft and hard states based on the standard state, MESIA exhibited a 10.55% increase in the soft state and a 20.47% decrease in the hard state. This method had the smallest amount of variation among all methods, except for NOMS like union method (Fig. 6A). To investigate the differences in the number of peaks based on the degree of reproducibility, we identified the regions where the peaks formed in each state using org.Hs.eg.db^27^ (v4.3). In the hard state, MESIA formed the fewest peaks in the distal intergenic region, while forming the most peaks in the promoter region when the region within 3 KB from the transcription start site was defined as the promoter region (Fig. 6B). This trend was consistent in both soft and standard states (Supplementary Fig. 1).

### Simulation results

**Figure 7:**
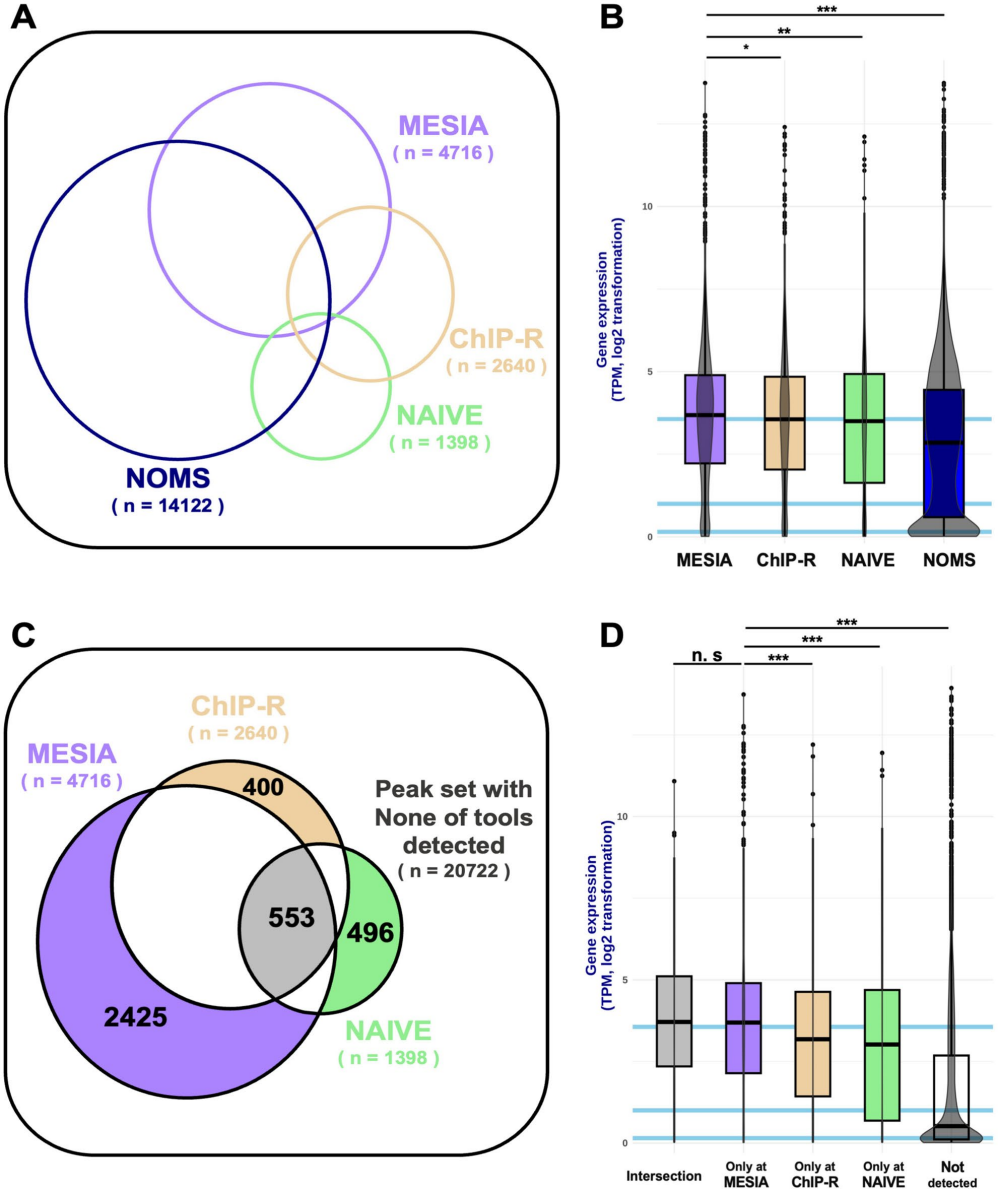
Simulation results. (**A**) Venn diagram of genes with optimal peaks formed in a promoter region across MESIA and compared algorithms. (**B**) Gene expression level of the genes identified by MESIA and compared algorithms. PolyA plus RNA-seq was utilized and it is expressed as a violin plot using log2 normalization gene expression. The sky-blue lines represent Q3, median, and Q1 in order, based on the expression levels of genes in the hg19 reference genome. (**C**) Venn diagram of genes which detected exclusively by each algorithm in MESIA, ChIP-R and NAIVE. (**D**) Gene expression level of the genes detected exclusively by each algorithm. (n.s., *p* > 0.05; *, 0.01 < *p* < 0.05; **, 0.001 < *p* < 0.01; ***, *p* < 0.0001).

We compared the performance of the methods depending on a degree of reproducibility. based on the three previously defined states. First, we present the distribution of genes in which optimal peaks were found in the promoter region for each method. In the hard state, for the other three algorithms that used the significant integration approach, MESIA identified 4,716 genes, which was 1.78 times more than ChIP-R and 3.37 times more than NAIVE (Fig. 7A). This trend was also observed for the soft and standard states (Supplementary Fig. 2).

We compared the performance of these algorithms in detecting genes based on gene expression levels. The expression of genes detected by MESIA was significantly different from that detected by all the comparative algorithms (*p* = 0.04402, *p* = 0.004593, *p* < 2.2e-16) (Fig. 7B). We present the distribution of genes detected by each significant integration approach algorithm using a Venn diagram. In the hard state, among the three algorithms, MESIA detected the highest number of genes (2425). This was 6.06 times higher than the number detected by ChIP-R and 4.88 times higher than that detected by NAIVE (Fig. 7C). The expression of genes detected only at MESIA was significantly different from that at ChIP-R and NAIVE (*p* = 4.142e-05 and *p* = 7.382e-09, respectively). (Fig. 7D). Also, we separately compared NAIVE and ChIP-R with MESIA. The trend of MESIA maintaining better performance has continued. (Supplementary Figs. 3 and 4). Similarly, to this approach, we compared the performance of the algorithms according to number of similar replications. In the weak state, for the three algorithms that used the significant integration approach, MESIA identified 1218 genes. This value is in the middle of those of the three algorithms (Supplementary Fig. 5A). The genes detected by MESIA were significantly different from those detected by all the other comparative algorithms. (*p* = 0.01707, *p* = 5.369e-05, *p* < 2.2e-16 in order). (Supplementary Fig. 5B). This trend was also observed for the strong and normal states (Supplementary Fig. 6). In the weak state, among the three algorithms, MESIA ranked in the middle for the number of genes detected exclusively by this method and the expression of genes detected only in MESIA was significantly different from that in ChIP-R and NAIVE (*p* = 0.04749 and *p* = 0.00295, respectively). (Supplementary Fig. 5C and D). MESIA's consistent improvement in performance has remained a prevailing trend. (Supplementary Figs. 7 and 8).

### Validation results

**Figure 8:**
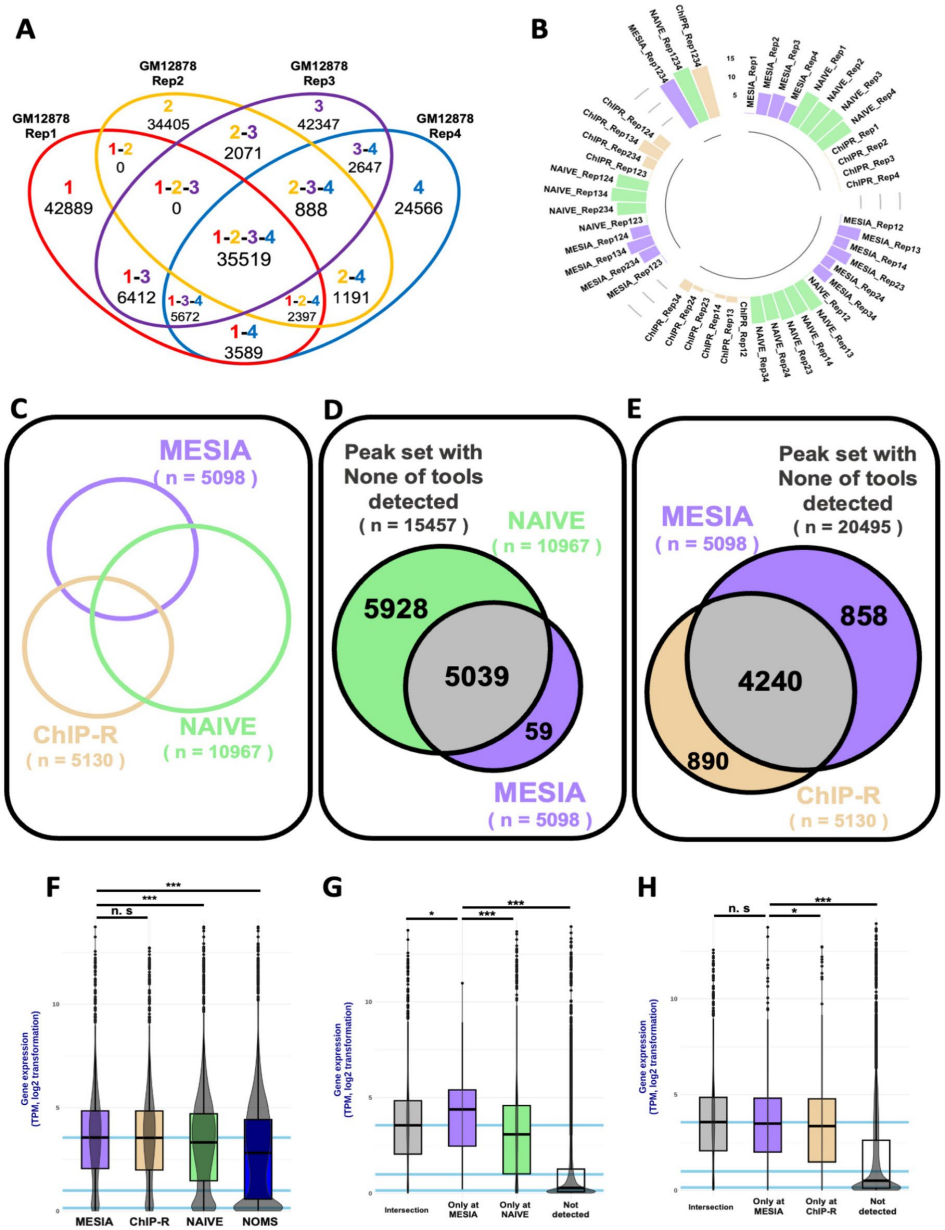
Validation Results. (**A**) Venn diagram of genes with optimal peaks formed in a promoter region across MESIA and compared algorithms. (**B**) Circular bar plot visualized number of replications related to peak. Circular bar plot visualized number of replications related to peak. (**C**) Venn diagram of genes with optimal peaks formed in a promoter region across MESIA and compared algorithms. (**D**) Venn diagram of genes with optimal peaks formed in a promoter region which detected exclusively by each algorithm in MESIA and NAIVE. (**E**) Venn diagram of genes with optimal peaks formed in a promoter region which detected exclusively by each algorithm in MESIA and ChIP-R. (**F**) Gene expression level of the genes identified by MESIA and compared algorithms. PolyA plus RNA-seq was utilized and it is expressed as a violin plot using log2 normalization gene expression. The sky-blue lines represent Q3, median, and Q1 in order, based on the expression levels of genes in the hg19 reference genome. (**G**) Gene expression level of the genes detected exclusively by each MESIA and NAIVE. (**H**) Gene expression level of the genes detected exclusively by each MESIA and ChIP-R. (n.s., *p* > 0.05; *, 0.01 < *p* < 0.05; **, 0.001 < *p* < 0.01; ***, *p* < 0.0001).

The performance was compared using real data. The distribution of the number of peaks in the four GM12878 replications is shown as a Venn diagram (Fig. 8A). For each method, the number of replicates related to each peak was classified and expressed as a circular bar plot. As expected, most peaks overlapped in all four replicates. Interestingly, MESIA showed the smallest difference in the peak according to the number of overlapping replications among the three compared methods. (Fig. 8B). MESIA found that replications 2, 3, and 4 were reproducible among the 4 replications. Therefore, it primarily identified regions where peaks were formed simultaneously in the 3 replications as optimal peaks. (Supplementary Fig. 10). The Venn diagram displays the gene distribution where each method formed optimal peaks in the promoter region. (Fig. 8C). We performed a comparison of the methods used to detect genes that showed significant differences in expression levels. The genes detected by MESIA were significantly different from those detected by NAIVE. (Fig. 8F) (*p* = 1.117e-13). To further analyze this difference, we conducted a comparison between only compared MESIA and NAIVE. We observed that 98.84% of the genes detected by MESIA overlapped with those detected by NAIVE. (Fig. 8D). The expression levels of genes detected by MESIA were higher than the Q3 threshold based on the expression levels of genes in the hg19 reference genome. Genes that were detected only by NAIVE and not by MESIA had expression levels lower than Q3, and this difference was statistically significant compared to the expression levels of genes detected only by MESIA. (Fig. 8G) (*p* = 0.0001482) We also compared MESIA with ChIP-R using a similar approach. The expression levels of genes detected using only MESIA were significantly higher than those detected using only ChIP-R. (*p* = 0.0352), respectively (Fig. 8E and H). These analysis results were also similar in mouse embryonic fibroblasts datasets^8^, which are not human datasets. Therefore, it was revealed that MESIA maintains good performance even in datasets that are not human lymphoblastoid datasets. (Supplementary Fig. 11). We evaluated the similarity between the analysis using the latest reference genome, hg38, and the analysis using hg19, employing the Jaccard similarity. The resulting value of 0.9743 was exceptionally high, indicating that the analyses using the two reference genomes produced virtually identical results.

## Discussion

The challenges of the diversity and integration of peaks discovered in multi-sample epigenomic analyses still exist. In this study, we proposed a new integrative method for multi-sample epigenomic analysis. This method combines epigenetic data from multiple samples to identify consistent peak patterns and will be valuable for genomic analysis of high-resolution genome and epigenome data.

Previous studies have shown that shared peaks across different cell types and tissues are associated with regulatory elements, such as enhancers and promoters, and play important roles in the regulation of gene expression^28, 29^. Our MESIA analysis identified conserved peak patterns associated with known regulatory elements, suggesting that our method can effectively identify biologically relevant peaks shared across multiple samples. Several studies integrated epigenomic signals from various disease models and computational approaches.

Epigenome peak analysis has emerged as a powerful tool for investigating the molecular mechanisms underlying common diseases, and numerous studies have used this approach to identify disease-associated epigenetic changes. Recent studies have used epigenomic peak analysis to identify novel therapeutic targets for Alzheimer’s disease, autism, and type2 diabetes. Researchers have identified unique peak patterns that are associated with the disease and used this information to develop a new diagnostic tool for disease^30–32^. Moreover, in cancer research, many researchers have used multi-sample epigenome peak analysis to identify epigenetic changes associated with cancer development and progression. Researchers have found that certain epigenetic modifications are more prevalent in cancer patients and have developed new therapeutic strategies targeting these modifications^33–35^.

There are several methods for integrating epigenomic peaks. For example, ChromVAR uses a latent variable model to identify shared and sample-specific regulatory elements across multiple epigenomic datasets. It can also perform differential analyses and prioritize disease-associated variants^36^. The scABC is designed for single-cell epigenomic data and uses a Bayesian clustering approach to identify clusters of cells with similar epigenomic profiles across multiple samples or conditions. It can be used to identify cell type-specific and shared regulatory elements, and to study cellular heterogeneity^37^. Moreover, Episom uses a multi-sample peak-calling approach to identify shared and sample-specific peaks across multiple epigenomic datasets. It can also be integrated with other genomic data types, such as RNA-seq and ChIP-seq, to identify candidate regulatory regions and their target genes^38^. EpiTensor uses a tensor decomposition approach to identify shared and sample-specific epigenomic patterns across multiple samples and conditions. They can be used to identify regulatory elements and gene regulatory networks that are conserved or variable across different contexts^39^.

Although some methods use statistical approaches, most use a length overlap-based approach. However, to the best of our knowledge, there are no methods that can integrate multiple samples while considering reproducibility between replicates. Specifically, most methods do not consider the reproducibility between replicates while integrating the peaks. Consequently, a statistical approach is vulnerable to noise caused by significant differences between samples. Our research aims to extract more accurate and reliable peaks by utilizing a statistical methodology that is more active than traditional multi-sample integration methods. To achieve this, we statistically extracted the significance of the individual peaks and integrated the peak information from multiple samples to determine whether each peak was significant. As a result, compared to other existing algorithms in situations where the degree of reproducibility is not sufficiently ensured, MESIA was able to detect peaks in up to 3.37 times more genes. Furthermore, the genes detected only by MESIA showed significantly higher expression levels than those detected by the other algorithms. In situations where the number of similar replications was insufficient, MESIA also identified genes that were significantly more highly expressed. These results show that MESIA, in addition to detecting genes with significantly higher expression by finding strong signals, also filters out false positive peaks that other algorithms miss. It does this by selecting peaks from reproducible replications. This methodology can be applied regardless of the sample size, allowing us to obtain valid results even with small sample sizes. To do this, appropriate statistical adjustments and simulations are necessary. For example, statistical adjustments were performed for all possible sample combinations and accurate results were obtained through simulations of individual combinations. As a result, we were able to obtain robust peaks that were not strongly affected by the degree of reproducibility and the number of similar replicates. Our study has some limitations. MESIA considers the reproducibility between replications before proceeding with Multi-Sample Integration, which may take a considerable amount of time and generate intermediate products proportional to the number of replications. As with all other methods, further validation using additional genomic data or experiments is necessary to increase the reliability of the results.

The integration of multi-sample epigenome peak analysis has various applications. One is in the development of new therapeutic strategies. Epigenetic modifications have been shown to play a critical role in the regulation of gene expression, and aberrant epigenetic changes have been implicated in the development and progression of various diseases. By identifying disease-specific peak patterns, researchers can develop new therapeutic strategies that target these epigenetic modifications. For example, drugs that target specific epigenetic enzymes or regulatory proteins can be developed to correct aberrant epigenetic changes and restore normal gene expression^40^.

MESIA is a user-friendly, open-source tool that enables researchers to integrate various epigenetic changes easily and accurately. By shedding light on the sophisticated regulation of gene expression and disease development, this approach can provide valuable and novel biological insights.

### Supplementary Information

Supplementary Information 1.

Supplementary Information 2.

Supplementary Information 3.

Supplementary Information 4.

Supplementary Information 5.

Supplementary Information 6.

Supplementary Information 7.

Supplementary Information 8.

Supplementary Information 9.

Supplementary Information 10.

Supplementary Information 11.

## Data Availability

ATAC-seq and RNA-seq datasets are publicly available on the NCBI Gene Expression Omnibus website (accession numbers GSE47753, GSE78554 and GSE145705).

## Code availability

Source codes for MESIA are freely available on GitHub (https://github.com/ERASMUSlab/MESIA).
